# *Notes from the Field:* Botulism Type E After Consumption of Salt-Cured Fish — New Jersey, 2018

**DOI:** 10.15585/mmwr.mm6844a3

**Published:** 2019-11-08

**Authors:** Pavan V. Ganapathiraju, Radhika Gharpure, Deepam Thomas, Natalie Millet, Daniel Gurrieri, Kevin Chatham-Stephens, Janet Dykes, Carolina Luquez, Perraju Dinavahi, Sandhya Ganapathiraju, Scott Roger, Danish Abbasi, Nancy Higgins, Frances Loftus, Manish Trivedi

**Affiliations:** ^1^AtlantiCare Regional Medical Center, Pomona, New Jersey; ^2^Epidemic Intelligence Service, CDC; ^3^Division of Foodborne, Waterborne, and Environmental Diseases, National Center for Emerging and Zoonotic Infectious Diseases, CDC; ^4^Communicable Disease Service, New Jersey Department of Health; ^5^Department of Critical Care AtlantiCare Regional Medical Center-Mainland Campus, Pomona, New Jersey; ^6^Coastal Infectious Disease Consultants, Galloway, New Jersey.

On October 25, 2018, at 2:15 a.m., a woman aged 30 years and her mother, aged 55 years, both of Egyptian descent, arrived at an emergency department in New Jersey in hypotensive shock after 16 hours of abdominal pain, vomiting, and diarrhea. The daughter also reported blurry vision and double vision (diplopia), shortness of breath, chest pain, and difficulty speaking. She appeared lethargic and had ophthalmoplegia and bilateral ptosis. Both women were admitted to the hospital. The mother improved after fluid resuscitation, but the daughter required vasopressor support in the intensive care unit. Although the mother did not have evidence of cranial nerve involvement on admission, during the next 24 hours, she developed dysphagia and autonomic dysfunction with syncope and orthostasis and was transferred to the intensive care unit as her symptoms progressively worsened similar to those of her daughter.

Two days before admission, both women had eaten fesikh, a traditional Egyptian fish dish of uneviscerated gray mullet that is fermented and salt-cured. Fesikh has been linked to foodborne botulism, including a large type E outbreak in Egypt in 1993 (*1*). The Egyptian Ministry of Health has since issued public health warnings regarding fesikh before Sham el-Nessim, the Egyptian holiday commemorating the beginning of spring, during which fesikh is commonly prepared and eaten.* Foodborne botulism outbreaks associated with fesikh and similar uneviscerated salt-cured fish have also occurred in North America (*2*); two outbreaks occurred among persons of Egyptian descent in New Jersey in 1992 (*3*) and 2005 (*4*)_._

Botulism, a paralytic illness caused by botulinum neurotoxin (BoNT), was suspected because of the reported exposure to fesikh along with symptoms of ophthalmoplegia, bilateral ptosis, dysarthria, and autonomic dysfunction. Per New Jersey Reporting Regulations (NJAC 8:57),^†^ these suspected illnesses were immediately reported to the New Jersey Department of Health. After consultation with CDC, heptavalent botulism antitoxin was released by CDC and administered to both patients within approximately 24 hours of arrival at the hospital. The daughter’s symptoms improved, and she was weaned off vasopressors. Both patients survived following intensive care for 2 days and total hospitalization of 7 days each.

CDC tested serum obtained before antitoxin administration. Serum from the daughter tested positive for BoNT type E by the BoNT Endopep-MS assay (*5*); the mother’s serum tested negative. A leftover sample of the consumed fesikh also tested positive for BoNT type E and *Clostridium botulinum* type E.

Interviews conducted by the Communicable Disease Service at the New Jersey Department of Health revealed that two fresh mullets purchased by the patients’ neighbor at a local Asian market were used to prepare the fesikh. The mother salt-cured and fermented the mullet, leaving the fish uneviscerated and wrapped in plastic in the kitchen for 20 days at ambient temperature. The mother confirmed that she previously used the same method of preparation in Egypt with no deviation in techniques or steps.

These cases illustrate the importance of early recognition and treatment of botulism. Botulism can be fatal, typically from respiratory failure, and treatment delays can result in increased mortality and worsened overall outcomes (*6*). These cases also highlight the role of uneviscerated, salt-cured fish dishes as potential vehicles for foodborne botulism. *C. botulinum* spores are ubiquitous in marine environments, and traditional methods of home preparation for these dishes might support conditions that are favorable for toxin production (i.e. anaerobic conditions) (*2*). Neither of these patients had previously heard of botulism. Risk communication via public awareness campaigns, as has been conducted by the Egyptian Ministry of Health to discourage fesikh consumption, might be indicated in the United States; engagement with Egyptian communities in the United States might provide insights into additional prevention strategies to decrease the risk for foodborne botulism from fesikh and other uneviscerated, salt-cured fish products.

## Footnotes

* https://www.nytimes.com/2013/05/07/world/middleeast/a-taste-of-spring-that-reeks-of-tradition.html.

† https://www.state.nj.us/health/cd/reporting/acode/index.shtml.

## References

[1] Weber JT, Hibbs RG Jr, Darwish A, A massive outbreak of type E botulism associated with traditional salted fish in Cairo. J Infect Dis 1993;167:451–4. 10.1093/infdis/167.2.451 8421179

[2] Food and Drug Administration. Uneviscerated fish products that are salt-cured, dried, or smoked (revised). CPG sec. 540.650. Silver Spring, MD: US Department of Health and Human Services, Food and Drug Administration; 2005. https://www.fda.gov/regulatory-information/search-fda-guidance-documents/cpg-sec-540650-uneviscerated-fish-products-are-salt-cured-dried-or-smoked-revised

[3] CDC. Outbreak of type E botulism associated with an uneviscerated, salt-cured fish product—New Jersey, 1992. MMWR Morb Mortal Wkly Rep 1992;41:521–2. 1630429

[4] Sobel J, Malavet M, John S. Outbreak of clinically mild botulism type E illness from home-salted fish in patients presenting with predominantly gastrointestinal symptoms. Clin Infect Dis 2007;45:e14–6. 10.1086/518993 17578769

[5] Barr JR, Moura H, Boyer AE, Botulinum neurotoxin detection and differentiation by mass spectrometry. Emerg Infect Dis 2005;11:1578–83. 10.3201/eid1110.041279 16318699 PMC3366733

[6] Sobel J. Botulism. Clin Infect Dis 2005;41:1167–73. 10.1086/444507 16163636

